# Novel phenotype of 5p13.3-q11.2 duplication resulting from supernumerary marker chromosome 5: implications for management and genetic counseling

**DOI:** 10.1186/s13039-018-0372-6

**Published:** 2018-03-27

**Authors:** Margaret E. Armstrong, David D. Weaver, Melissa D. Lah, Gail H. Vance, Benjamin J. Landis, Stephanie M. Ware, Benjamin M. Helm

**Affiliations:** 1 Department of Medical and Molecular Genetics and Department of Pediatrics, Indiana University School of Medicine, 550 N. University Blvd, AOC 5001, Indianapolis, Indiana 46202 USA; 2 Department of Pediatrics, Indiana University School of Medicine, Indianapolis, Indiana USA; 3 Department of Internal Medicine, University of Iowa Hospitals and Clinics, Iowa City, Iowa USA

**Keywords:** Supernumerary marker chromosome, Chromosome 5, Genotype-phenotype correlation, Aortic aneurysm, 5p13 duplication

## Abstract

**Background:**

Supernumerary marker chromosomes derived from chromosome 5 (SMC5) and 5p13 duplication syndrome are rare disorders, and phenotypic descriptions of patients are necessary to better define genotype-phenotype correlations for accurate, comprehensive genetic counseling. The purpose of this study is to highlight the unique findings of a patient with a 5p13.3-q11.2 duplication arising from a SMC5 and compare and contrast the phenotype with cases in the literature.

**Case presentation:**

We report on an adult male with a 22 Mb duplication of chromosome 5p13.3-q11.2 resulting from a small SMC5. The patient has a history of prenatal polyhydramnios, dysmorphic features, respiratory issues, talipes equinovarus, hypotonia, developmental delay, and autistic features. The patient also has novel features of aortic dilation, pectus excavatum, kyphoscoliosis, and skin striae, suggestive of a connective tissue disorder. Despite these features he did not meet clinical diagnostic criteria for a well-characterized connective tissue disorder. Additional molecular genetic testing for syndromic and non-syndromic aortic aneurysms was negative.

**Conclusions:**

Many of the patient’s features are consistent with individuals reported with 5p13 duplication syndrome and similar cases of SMC5, including polyhydramnios, macrocephaly, dolichocephaly, pre-auricular pits, arachnodactyly, respiratory problems, and developmental delays. It is unclear if the patient’s unique features of aortic dilation, pectus excavatum, kyphoscoliosis, and skin striae could be novel features of the SMC5 given its rarity and the few well-phenotyped adults in the literature. This report reviews the literature and provides additional phenotypic information to define the genotype-phenotype correlation of SMC5 and 5p13 duplication syndrome.

## Background

A supernumerary marker chromosome (SMC) is a structurally abnormal chromosome with ambiguous chromosomal origin [1]. An SMC can originate from any chromosome and occurs in approximately 0.044% of all newborns [2, 3]. SMCs are most commonly derived from chromosomes 15 and 22, and it is estimated that 70% of SMCs are de novo*,* while 30% are inherited [1, 4]. There are a small number of syndromes associated with well-characterized SMCs, such as Emanuel, Pallister-Killian, and cat-eye syndromes [2, 5]. Cases of SMCs derived from chromosome 5 (SMC5) are rare and make up 1.4% of all reported and characterized SMC cases [6]. In these cases, the most common features reported are macrocephaly, dysmorphic facial features, heart defects, growth retardation, hypotonia, and intellectual disability [4].

The phenotype resulting from a particular SMC ranges from normal to severely abnormal based on the chromosome origin, size, euchromatin content, and level of mosaicism [4, 7]. An SMC can result from various mechanisms in meiosis and mitosis, including double-stranded DNA breaks, telomere-subtelomere junction, and an inverted duplication associated with a deletion [8]. Because of these variables, the phenotypic effects of SMCs are difficult to catalog and only one-third of SMCs are associated with a unique clinical picture [1, 9]. Presently, approximately half of all SMCs have a ring chromosome morphology, in which the two ends of the chromosome fragment have fused together to form a ring [10]. It was previously reported that greater than half of ring chromosome SMCs are associated with an abnormal phenotype [9, 11].

The de novo inheritance and heterogeneity of SMCs pose a challenge for genetic counseling and medical management. Information regarding the size, gene content, and clinical presentation resulting from SMCs facilitates predictions regarding the potential clinical effects and prognosis, i.e., genotype-phenotype correlation associated with SMCs. In the present study, we report a patient with an SMC derived from chromosome 5 with a chromosomal microarray result revealing a 22 Mb duplication of chromosome 5p13.3-q11.2. Based on the Small Supernumerary Marker Chromosome Database (http://ssmc-tl.com/sSMC.html), there no other cases reported with this specific duplication [12]. We review and compare this case with other cases with overlapping content of SMCs composed of chromosome 5 material.

## Case presentation

**Fig. 1:**
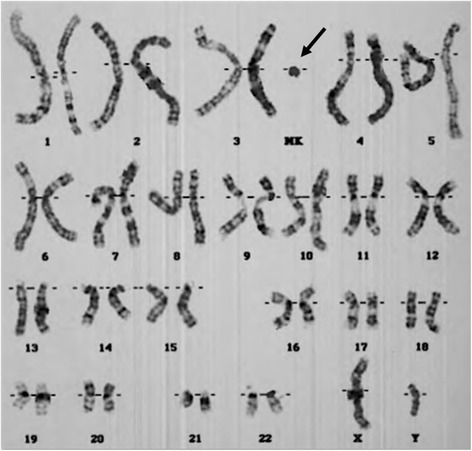
A G banded karyogram was completed in 1997. The unidentified SMC is indicated by the arrow. Karyotype: 47,XY,+r [15]/46,XY [17]

The patient was born at 30 weeks gestation to a 26-year-old G1P0 mother following a pregnancy complicated by polyhydramnios and preterm labor occurring at 24 weeks gestation. At birth the patient’s weight was 1.786 kg (90th centile) and the length was 43 cm (90th centile). After delivery, he was noted to be dysmorphic and have right talipes equinovarus. At infancy, head imaging was completed showing polymicrogyria; however, the clinical team was unsuccessful in obtaining original images from these scans and follow-up imaging in adulthood was not completed. Fluorescence in situ hybridization (FISH) testing for chromosome 17p deletion associated with Miller-Dieker syndrome was negative; this test was performed during infancy. An initial chromosome analysis (karyotype) on peripheral blood after birth revealed the presence of 40–45% mosaicism for a SMC with possible ring structure (Fig. 1). A second karyotype repeated during infancy identified the SMC in each of the cells analyzed, though its structure was unclear given its size and the decreased resolution of the analysis. These chromosome analyses were performed by different laboratories and interpreted by independent clinical cytogeneticists. Follow-up FISH multi-probe testing revealed that the SMC was composed of chromosome 5 material. Before being discharged, the patient spent 5 weeks in the hospital because of respiratory problems. He was released on an oxygen saturation monitor, caffeine, mini nebulizer treatments, and a feeding tube.

As an infant, the patient had low muscle tone and his developmental milestones were globally delayed. He sat by himself at 10 months, crawled at 12 months, and walked at 18 months. He received occupational, physical, speech, and developmental therapies. Additionally during childhood, the patient’s medical history was significant for dental caries, strabismus, myopia, kyphoscoliosis, and chronic diarrhea. He was also diagnosed with autism spectrum disorder and attention deficit-hyperactivity disorder (ADHD).

After being lost to follow-up care during childhood and adolescence, the patient was referred for a clinical genetics evaluation at age 18 due to severe scoliosis and concerns by his cardiologist for a connective tissue disorder. The patient’s kyphoscoliosis was at a Risser stage 4 and required spinal fusion at age 18 years. During that same year, a heart murmur was heard and an echocardiogram revealed mild aortic root dilation with a sinus of Valsalva diameter of 3.60 cm (Z-score: + 2.3) and ascending aorta diameter of 3.38 cm (Z-score: + 2.8). He subsequently was prescribed treatment with beta blockers. The patient was then seen by our clinical genetics team, and at this visit the patient had chromosome microarray analysis and molecular genetic testing performed on peripheral blood.

A four-generation family history was significant for a maternal great-grandfather dying from complications of a thoracic or abdominal aortic aneurysm, though details were unclear. The patient’s parents are alive and well without any significant health history. No cardiac imaging has been completed in family members. The remainder of the patient’s family history is unremarkable for birth defects, intellectual disability, or recurrent miscarriages. Consanguinity was denied.

On physical examination the patient had a height of 185 cm (87th centile), weight of 81 kg (80th centile), arm span to height ratio of 1.0, bilateral pre-auricular pits, kyphoscoliosis, pectus excavatum, three vertical skin striae on the back, and dysmorphic head and facial features including head circumference of 61.5 cm (>97th centile), dolichocephaly, a long and thin face, hypotelorism with an inner canthal distance of 2.9 cm (<3rd centile), mild malar hypoplasia, esotropia, and dental crowding. He had a Beighton score of 0 out of 9 and a systemic score of 4 by the revised Ghent criteria based on the presence of positive bilateral wrist signs, pectus excavatum, kyphoscoliosis, and skin striae. He did not meet clinical criteria for a diagnosis of a connective tissue disorder such as Marfan, Loeys-Dietz, or Ehlers-Danlos syndromes.

## Methods

The chromosome analyses and FISH multi-probe test were completed on blood samples during infancy by an outside institution. A chromosomal microarray analysis (CMA) and thoracic aortic aneurysm and dissection (TAAD) gene panel were completed through GeneDx (Gaithersburg, Maryland). Each of these tests was completed using common commercial preparation and analytical methods. The CMA was performed using a combination copy-number and SNP array. The CMA was performed using a combination copy-number and SNP array custom designed by GeneDx (v5). This is a targeted and whole genome backbone array with 118,000 copy number probes and 66,000 SNP probes. The resolution across the backbone (genome) is ~ 200 kb and ~ 500 bp to 15 kb within the targeted regions. The targeted regions typically contain multiple markers associated with known genetic syndromes.

The TAAD gene panel analyzed 16 genes (*ACTA2, CBS, COL3A1, COL5A1, FBN1, FBN2, FLNA, MED12, MYH11, SKI, SLC2A10, SMAD3, TGFB2, TGFBR1, TGFBR2*), which were sequenced by massively parallel (NextGen) sequencing on an Illumina platform with paired-end reads. Bi-directional sequence was assembled, aligned to reference gene sequences based on human genome build GRCh37/UCSC hg19, and analyzed for sequence variants. Concurrent deletion/duplication testing was performed for the genes in the panel except *TGFB2, SKI, FLNA*, and *MED12* using exon-level oligo-array analysis (ExonArrayDx).

To review results of the CMA, the clinical team utilized the Genomic Oligoarray and SNP Evaluation Tool V3.0 to assess this genomic region. Intersection of the proband’s phenotype with genes in the region associated with disease was performed [13]. Focus was placed on genes related to aortic diseases and connective tissue biology/disease pathways given the patient’s reported phenotype [13]. Emphasis was placed on genes with currently known function and/or associations with disease using the Online Mendelian Inheritance in Man (OMIM) compendium.

## Results

**Fig. 2:**
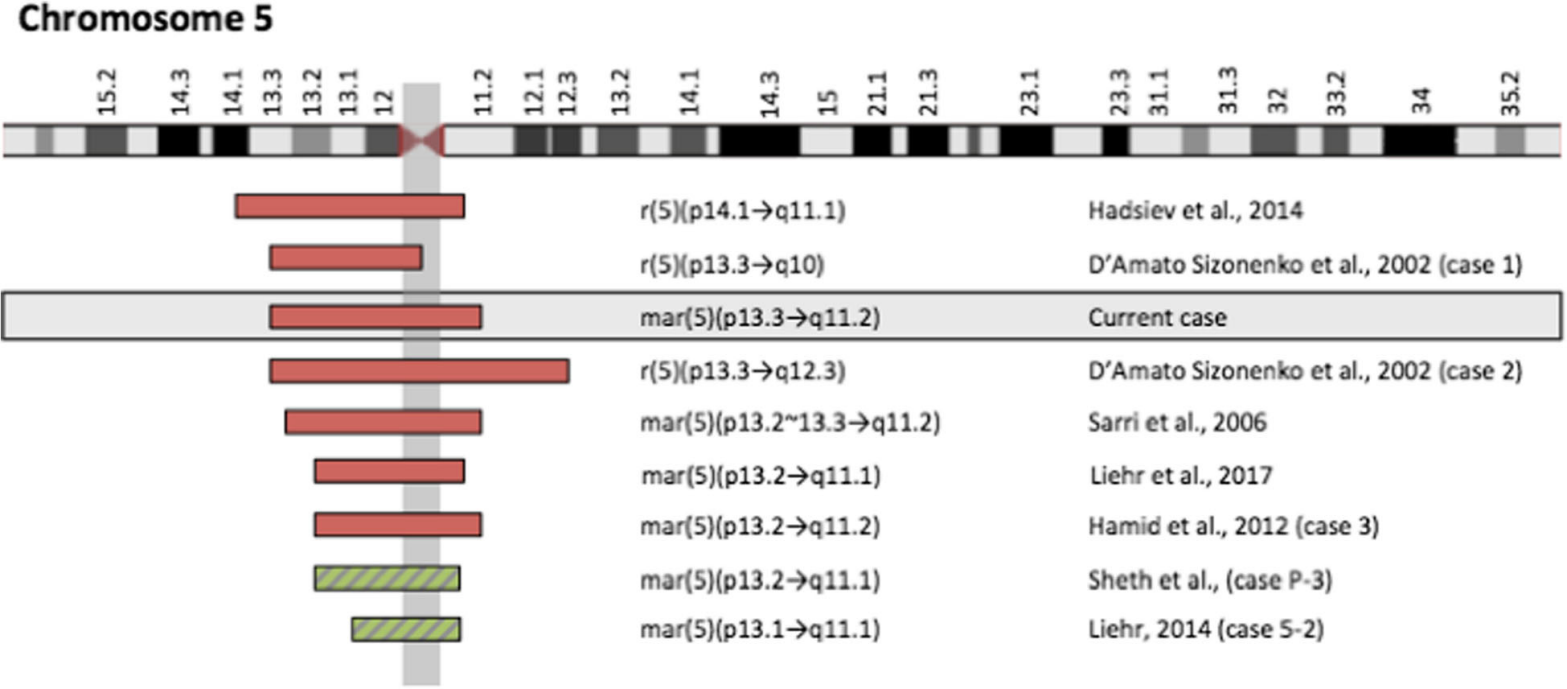
Cases of SMC5 overlapping the genomic coordinates of our patient. A diagrammatic representation of the chromosomal content involved in various cases from the Small Supernumerary Marker Chromosome Database (http://ssmc-tl.com/sSMC.html). These cases involve overlapping chromosome 5 material with our patient [6, 14, 22, 28–30]. The red boxes correspond to cases reported with clinical findings and the green boxes correspond to cases reported with negative clinical findings

The CMA identified a 22 Mb duplication of chromosome 5p13.3-q11.2 [hg19:31513816–53,752,188]. Using the genome browser detailed in the Methods section, there were 195 genes located in this region (of which 68 are currently annotated in OMIM). Based on the Small Supernumerary Marker Chromosome Database (http://ssmc-tl.com/sSMC.html), there are other cases reported with SMCs of overlapping chromosomal material (Fig. 2); however, no cases are reported with the same specific duplication [6]. The initial karyotype described the SMC as having a ring structure, though the structure was unclear in the second karyotype performed during early infancy. This is likely due to differences in interpretation by different institutions/cytogeneticists. The TAAD gene panel was negative and did not identify any pathogenic or likely pathogenic variants; additionally, no suspicious variants of unclear significance were identified.

## Conclusions

In this paper, we report on a case with an SMC composed of chromosome 5 material that was characterized by CMA and FISH testing. Our patient has a medical history significant for polyhydramnios, dysmorphic features, dental caries, respiratory issues, chronic diarrhea, talipes equinovarus, hypotonia, developmental delay/intellectual disability, autism spectrum disorder, and ADHD. Additionally, he has features overlapping with connective tissue disorders including mild aortic dilatation, pectus excavatum, kyphoscoliosis, and skin striae.

We reviewed the literature for additional cases of SMCs involving overlapping chromosome 5 material. The individuals in these cases possess features that vary widely (Table 1) [6, 7, 10, 14, 15]. The Small Supernumerary Marker Chromosome Database (http://ssmc-tl.com/sSMC.html) cites multiple case reports of SMCs involving chromosome 5 material, which overlap the genomic coordinates of our patient (Fig. 2) [6]. Ten individuals were reported to have an SMC of 5p11-q11.1, predominantly involving the pericentromeric region. These individuals were reported not to have any clinical findings; however, three of the four adult male patients did report experiencing fertility problems [6]. The pericentromeric region of chromosome 5 is suggested to be non-dosage sensitive, whereas the chromosomal regions located further from the centromere are considered to be dosage sensitive [6]. Within individuals who were described to have clinical findings, some of the most common features were dysmorphic facial features, congenital heart defects involving ventricular septal defects and atrial septal defects, talipes equinovarus, failure to thrive, hypotonia, developmental delay, and intellectual disability (Table 1). Additionally, two cases reported by D’Amato Sizonenko et al. [14] and one by Avansino et al. [15] described individuals with SMC’s from chromosome 5 who experienced polyhydramnios during pregnancy, which was also reported in our patient.

**Table 1:** The major clinical features of individuals with overlapping SMC5’s to our patient

SMC5 Coordinates	p10 → p13.1 (Avansino et al., 1999 [15])	p14 → q11.2 (Stankiewicz et al., 2000 [31])	p10 → p13.3 (D’Amato Sizonenko et al., 2002 [14])	p13.3 → q12.3 (D’Amato Sizonenko et al., 2002 [14])	p12 → q10 (Liehr et al., 2006 [9])	p13.2 → q11.2 (Sarri et al., 2006 [22])	p11 → q12.1 (Baldwin et al., 2008 [10])	p11 → p13.3 (Loscalzo et al., 2008 [21])	p11 → q12.1 (Melo et al., 2011 [7])	p14 → q11.1 (Hadzsiev et al., 2014 [28])	p13.2 → q11.2 (Liehr, 2016 [12])	p13.2 → q11.1 (Liehr, 2016 [12])	p13.2 → q12.2 (Camerota et al., 2017 [32])	p13.3 → q11.2 (Current Case)
Age at Evaluation	5 months	27 years	3 months	9 months	1 week	9 years	1 year	5 years	4 years	10 years	4 years	10 months	17 years	18 years
Dysmorphic^a^ Facial Features	+	+	+	+	+	+	+	+	+	+	+	+	+	+
Pectus Excavatum	–	–	–	–	–	–	–	–	–	+	–	–	–	+
Respiratory Issues	+	–	+	+	–	–	+	+	–	–	–	–	–	+
Congenital Heart Defect	+	–	–	+	+	+	+	+	–	–	–	+	–	–
Aortic Dilation	–	–	–	–	–	–	–	–	–	–	–	–	–	+
Scoliosis	–	–	–	–	–	–	–	–	–	–	–	–	–	+
Talipes Equinovarus	–	+	+	+	+	–	–	–	–	–	–	–	–	+
Skin Striae	–	–	–	–	–	–	–	–	–	–	–	–	–	+
Hypotonia	+	NR	+	+	+	–	–	+	+	+	+	–	–	–
Seizures	–	–	+	–	–	–	–	+	–	–	–	–	–	–
Developmental Delay	+	NR	+	+	+	–	+	+	+	+	+	–	–	+
Speech Delay	NR	+	+	–	+	–	–	+	+	+	+	–	–	–
Intellectual Disability	NR	+	–	–	–	NR	–	NR	+	+	+	–	–	+
Failure to Thrive	+	NR	+	+	–	–	+	+	–	–	–	–	–	–

^a^Dysmorphic facial features described in individuals included dolichocephaly, epicanthal folds, upslanting palpebral fissures, hypertelorism, microphthalmia/coloboma, strabismus, broad nasal bridge, short nose, midface hypoplasia, macroglossia, microretrognathia, pre-auricular pits, and/or low-set/dysplastic ears [6, 7, 10, 14, 15, 22, 28, 31, 32]. “NR” represents that the feature was not reported in the publication

The clinical features described in the patient reported here are concordant with many of the features reported in other cases with similar SMC, which strengthens a genotype-phenotype correlation for the patient’s SMC. Our patient does have additional features of aortic dilation, pectus excavatum, kyphoscoliosis, and skin striae that are not reported in the other cases of SMCs derived from chromosome 5. On physical examination our patient did not meet clinical criteria for a diagnosis of a specific connective tissue disorder and has had a negative TAAD gene panel with comprehensive coverage of 16 genes associated with these disorders. TAAD disorders are primarily autosomal dominant conditions with high penetrance. Further, the de novo mutation rate may be relatively high; e.g. Marfan syndrome’s rate is approximately 25% [16]. There was no evidence in the patient’s first-degree relatives of a connective tissue condition or known aortic disease. The patient’s connective tissue disorder features could be the result of a de novo mutation in a gene or gene region not currently testable by molecular genetic testing. It is also possible that these features could be a result of the patient’s chromosome anomaly, though we cannot rule out that this patient has two co-occurring conditions (the SMC5 and an unidentified genetic TAAD disorder). Other cases with similar SMC’s are not reported to have these features, however individuals with thoracic aortic aneurysm tend to be asymptomatic until late in the progression of the disease and typically have an older age of onset.

Past reports of similar SMC’s described infants and young children (a time period in which penetrance of aortic disease is low), and there are few adults reported in the literature. Additionally, previous reports did not perform serial cardiac imaging specifically for aortic dilation. Because of these points the presence of an older-onset, mildly penetrant aortic disease risk cannot be completely ruled out for similar SMC’s. It is unknown if aortic dilation could be an age-dependent feature of similar chromosome abnormalities. Therefore, some affected individuals may have gone undetected due to the absence of clinical indications for evaluation and a younger age at evaluation (Table 1). There are few adult SMC5 patients with clinical phenotypes detailed in the literature. Our patient’s unique features of a connective tissue disorder might also represent a novel phenotype in light of a novel SMC chromosome disorder, and individuals with similar SMC5 might benefit from cardiac evaluation for aortic dilation and treatment with beta blockers if dilation is present, significant, and/or progressive.

Our patient’s unique features could potentially be due to a dosage-gain effect of a particular gene(s) located within the SMC. According to the Genomic Oligoarray and SNP array evaluation tool v3.0 [13], the patient’s SMC contains 68 OMIM genes. It was reported that 25 of these genes are associated with specific disorders and ten of these are inherited in an autosomal dominant pattern. One significant gene in this region is *NIPBL,* and this gene is important for neurodevelopment*.* The *NIPBL* gene is located at 5p13.2 and encodes the delangin protein, which plays a role in human development. Pathogenic mutations within the *NIPBL* gene cause haploinsufficiency and are associated with more than half of Cornelia de Lange syndrome cases [17, 18]. Individuals with this condition typically have developmental disorders and behavioral problems. Duplications of regions on chromosome 5 containing this gene are also reported in individuals with developmental delays and other significant developmental or morphological features [19, 20]. Loscalzo et al. [21] proposed that a duplication of *NIPBL* could cause the congenital heart defects and seizures observed in some cases of chromosome 5p13 duplication. These authors also stated that a potential critical region on the p arm of chromosome 5 lies between 5p13.1 and 5p13.3, where the *NIPBL* gene is located [21].

We also reviewed the literature for cases of chromosome 5p13 duplication syndrome. These duplications are associated with dysmorphic facial features, developmental delays, learning disability, behavioral problems, and polyhydramnios [14, 15, 18, 21, 22]. Novara et al. [18] described a patient with a 5p13 duplication of at least 264 kb that only included the *NIPBL* gene. This patient’s features overlapped with chromosome 5p13 duplication syndrome and contributed to a better-defined genotype-phenotype correlation and confirmed the involvement of *NIPBL* in the features of 5p13 duplication syndrome [18]. The patient reported by Novara et al. had similar features to our patient, including dysmorphic facial features of malar hypoplasia and bitemporal narrowing, developmental delay, speech delay, and hypotonia [18]. It is interesting to note the report of malar hypoplasia in the case from Novara et al. since it is a craniofacial difference seen in cases of Marfan syndrome and Loeys-Dietz syndromes (though not in an exclusive fashion), suggesting that perhaps similar SMC5 cases had some overlapping features of connective tissue disorder.

A patient reported by Avansino et al. [15] was suspected to be the first reported case of 5p13 duplication syndrome associated with a marker chromosome. This patient has features similar to our patient, including polyhydramnios, macrocephaly, dolichocephaly, pre-auricular pits, arachnodactyly, respiratory problems, and developmental delays (Table 2) [15]. The patient also had an echocardiogram after birth revealing a perimembranous ventricular septal defect. Another patient reported by Sarri et al. [22] has a ring chromosome characterized as 5p13.2~ 13.3-q11.2, which is very similar to the genetic content within our patient’s SMC. Concordant features with our patient include macrocephaly, dolichocephaly, pre-auricular pits, arachnodactyly or tapering fingers, and some developmental delays [22]. The arachnodactyly feature is also interesting given its presence in many cases of Marfan syndrome and other connective tissue disorders. The patient completed cardiac imaging after birth due to an atrial and ventricular septal defect, but no subsequent imaging was reported. Overall, the clinical features in our patient are concordant with many of the features in other cases of 5p13 duplication syndrome, which helps to define the phenotype of this condition. However, our patient’s features of aortic dilation, pectus excavatum, kyphoscoliosis, and skin striae continue to be unique amongst cases of 5p13 duplication syndrome.

**Table 2:** Phenotypic overlap between our patient and a patient reported by Avansino et al. [15] and Sarri et al. [22]. Note some of the craniofacial and other physical features also reported in connective tissue disorders, e.g. dolichocephaly, malar hypoplasia/midface hypoplasia, etc. Some of these features were reported in Novara et al. [18] which is not included in this table

	Current Case	Avansino et al., 1999	Sarri et al., 2006
Duplication:	mar(5)(p13.3-q11.2) [40–45% mosaicism]	mar(5)(p10-p13.1)	r(5)(p13.2~ 13.3-q11.2) [60% mosaicism]
Dysmorphic Facial Features:			
Macrocephaly	**+**	**+**	**+**
Dolichocephaly	**+**	**+**	**+**
Epicanthal folds	**–**	**+**	**+**
Upslanting palpebral fissures	**–**	**+**	Down-slanting
Hypotelorism/ Hypertelorism	Hypo	Hyper	Hyper
Esotropia/strabismus	**+**	**–**	**–**
Short nose	**–**	**+**	**–**
Malar hypoplasia	**+**	**–**	**–**
Midface hypoplasia	**–**	**+**	**–**
Pre-auricular pit	Bilateral	Unilateral	Bilateral
High arched palate	**–**	**+**	**–**
Arachnodactyly/tapered fingers	**+**	**+**	**+**
Other Features:			
Polyhydramnios	**+**	**+**	**–**
Pectus excavatum	**+**	**–**	**–**
Respiratory issues	**+**	**+**	**–**
Congenital heart defect	**–**	**+**	**+**
Aortic dilation	**+**	NR	–
Scoliosis	**+**	**–**	**–**
Talipes equinovarus	**+**	**–**	**–**
Seizures	**–**	**–**	**–**
Hypotonia	**–**	**+**	**–**
Developmental delay	**+**	**+**	Borderline

*NR* not reported

The patient’s phenotype was intersected with genes known to function in vascular/endothelial and connective tissue biology. This was especially important given the patient’s novel connective tissue features including aortic dilation. Of the 68 OMIM genes in the SMC5, there was one gene that functions in connective tissue pathways, the *ADAMTS12* gene. This gene is a member of the large ADAMTS family of zinc-dependent proteases, specifically disintegrin and matrix metalloproteinases, known to have important roles in diverse pathological processes, like inflammation, cancer, and arthritis. These proteins also have roles in angiogenesis and atherosclerosis [23, 24]. Members of this protein family degrade components of the extracellular matrix, potentially influencing vascular disease occurrence and presentation. *ADAMTS12* was found function as a negative regulator of angiogenesis and vascular endothelial growth factor (VEGF) in animal endothelial cells [23]. Genetic variants in matrix metalloproteinases have been proposed to be involved in vascular pathology, including abdominal and thoracic aortic aneurysms. Previous studies have indicated that increased expression of *ADAMTS12* (and other ADAMTS genes) influenced disease by increasing degradation of components of the extracellular matrix [25]. While this possible disease process remains unconfirmed in our patient, it raises potential research questions/hypotheses regarding increased dosage of *ADAMTS12* due to the SMC5 and possible risk for aortic disease with age in similar cases. This remains to be explored in present and future cases of SMC5/5p13 duplications involving this gene.

In the present case, the patient has not had any other comprehensive genetic testing, such as clinical exome sequencing. In the absence of whole-exome sequencing, we are unable to rule out the presence of a de novo mutation in a gene or gene region not detected by the TAAD gene panel that could potentially be associated with our patient’s connective tissue disorder features. Please refer to Table 3 for a list of genes analyzed for the present case.

**Table 3:** Thoracic aortic aneurysm/dissection (TAAD) associated genes analyzed in the present case. Next-generation sequencing was performed for the following 16 genes; deletion/duplication analysis was completed in 12/16 genes per protocol by the commercial genetic testing laboratory (GeneDx, Gaithersburg, MD)

Gene		Analytical Methods	
		Sequencing	Deletion/Duplication
*ACTA2*	Multisystem smooth muscle dysfunction syndrome	**√**	**√**
*CBS*	Homocystinuria	**√**	**√**
*COL3A1*	Ehlers-Danlos syndrome type IV	**√**	**√**
*COL5A1*	Ehlers-Danlos syndrome type I	**√**	**√**
*FBN1*	Marfan syndrome	**√**	**√**
*FBN2*	Congenital contractural arachnodactyly	**√**	**√**
*FLNA*	Ehlers-Danlos syndrome and periventricular nodular heterotopia, X-linked cardiac valvular dysplasia	**√**	–
*MED12*	FG syndrome, Lujan syndrome, X-linked Ohdo syndrome	**√**	–
*MYH11*	Patent ductus arteriosis	**√**	**√**
*SKI*	Shprintzen-Goldberg syndrome	**√**	–
*SLC2A10*	Arterial tortuosity syndrome	**√**	**√**
*SMAD3*	Loeys-Dietz syndrome	**√**	**√**
*TGFB2*		**√**	–
*TGFBR1*		**√**	**√**
*TGFBR2*		**√**	**√**

The presence of a mosaic karyotype has been detected in over half of all SMC cases [9]. Prior karyotype analysis detected 40–45% mosaicism; however, there was no evidence for mosaicism based on our patient’s CMA results. Testing from a single cell type (white blood cells) cannot rule out mosaicism in other tissues and current CMA technologies are capable of detecting mosaicism of ≥25–37%, though could be as low as ≥10% [26, 27]. The varying number of abnormal cells in specific tissues could potentially impact the clinical expression of the extra chromosomal material.

As technology has advanced, our ability to identify the chromosomal content of SMCs has improved. Therefore, defining a genotype-phenotype relationship for SMCs will improve and will provide valuable information for genetic counseling, particularly with regard to prenatal diagnosis. The novel features reported here and the phenotype of an adult patient have been rarely reported in the literature. This information will contribute to our understanding of SMC5 and 5p13 duplication syndrome as well as serve as a resource for counseling patients and families in the future. Overall, the case presented here provides useful clinical information to formulate and strengthen a genotype-phenotype correlation for SMCs consisting of chromosome 5 material and to better define the phenotype for chromosome 5p13 duplication syndrome.

## Abbreviations

ADHD: Attention deficit-hyperactivity disorder
CMA: Chromosomal microarray analysis
FISH: Fluorescence in situ hybridization
OMIM: Online Mendelian Inheritance in Man
SMC: Supernumerary marker chromosome
TAAD: thoracic aortic aneurysm and dissection
